# Anomalous Left Main Coronary Artery in a Young Athlete: A Rare Cause of Sudden Cardiac Death

**DOI:** 10.7759/cureus.89209

**Published:** 2025-08-01

**Authors:** Kha Cong Van, Mark Gustafson, Caitlin Thomas, Amy E Hunt

**Affiliations:** 1 Emergency Department, West Virginia School of Osteopathic Medicine, Lewisburg, USA; 2 Emergency Medicine Department, Charleston Area Medical Center, Charleston, USA

**Keywords:** anomalous left main coronary artery, coronary artery angiography, coronary artery anomalies origin, right coronary sinus, sudden cardiac death (scd)

## Abstract

Sudden cardiac death (SCD) in young individuals raises serious concern, prompting physicians to explore multiple etiologies. Coronary artery anomalies are rare congenital heart defects, affecting a small percentage of the population, and are a known cause of SCD in young individuals. We present a case of sudden cardiac arrest in a 36-year-old male athlete with an anomalous left main coronary artery (ALCA) arising from the right coronary sinus, a rare anomaly that has been associated with SCD in young athletes during intense exertion. This cause underscores the importance of considering congenital artery anomalies in young patients presenting with SCD.

## Introduction

Coronary artery anomalies are rare congenital variants of coronary artery origin or course, affecting approximately 1% of the population [1]. They are often discovered incidentally during computed tomography (CT), coronary CT angiography (CCTA), or autopsy following sudden cardiac death (SCD). While most coronary artery anomalies are benign and are not associated with potentially serious sequela, hemodynamically significant anomalies, such as ectopic coronary artery origins from the pulmonary artery or opposite aortic sinus, can lead to myocardial ischemia, malignant arrhythmias, or SCD [2]. Anomalous left main coronary artery (ALCA) originating from the right coronary sinus, as observed in this case, is particularly associated with SCD in young athletes during intense exertion [1].

In a study at the Cleveland Clinic over 28 years, 1,686 of 126,595 patients (1.3%) who underwent coronary arteriography were found to have coronary artery anomalies, with 81% classified as benign [2]. ALCA from the right coronary sinus is rare (prevalence 0.0024%-0.02% in adults) and carries a high risk of adverse cardiac events [3,4]. While ALCA originating from the right coronary sinus presenting with chest pain and ischemic changes has been previously reported, our case adds to this literature by reporting survival from SCD of an athlete with ALCA originating from the right coronary sinus that ran interarterially between the ascending aorta and pulmonary trunk [4].

## Case presentation

A 36-year-old male presented to the emergency department via air emergency medical service (EMS) after a witnessed cardiac arrest during a marathon race. Bystanders noted that there had been a swarm of bees near the patient at the time of the cardiac arrest, causing initial concern for anaphylaxis. The patient was pulseless, and Advanced Cardiac Life Support (ACLS) protocol was performed at the scene with the use of an automated external defibrillator (AED). After three defibrillation attempts, the patient had a return of spontaneous circulation (ROSC). Paramedics intubated the patient in the field and administered intramuscular epinephrine and diphenhydramine presuming anaphylaxis despite the patient not having urticaria, stridor, or other signs of Hymenoptera envenomation. The patient’s past medical, family, and social history were unavailable.

Upon arrival at the emergency department, vital signs included a temperature of 36.3 °C, blood pressure 187/78 mmHg, heart rate 84 beats per minute, respiratory rate 22 breaths per minute, and oxygen saturation 100% on mechanical ventilation. Physical examination revealed bilateral lung rales, movement of all extremities, and sluggish but reactive 3 mm pupils. No urticaria or bee stings were noted. Several bags of electrolyte replacement pills were found on his person. Intravenous (IV) fentanyl and propofol drips were started for sedation, in addition to 4 mg of IV midazolam. He was additionally administered 125 mg IV methylprednisolone and 25 mg IV famotidine, given the prehospital concern for possible anaphylaxis from bee stings. Two liters of normal saline were also administered.

Laboratory results showed a respiratory acidosis, leukocytosis, and an elevated troponin (Table 1). 

**Table 1:** Initial laboratory evaluation from the Emergency Department. pH, blood pH level; pCO₂, partial pressure of carbon dioxide; pO₂, partial pressure of oxygen; HCO₃, bicarbonate; CO₂, carbon dioxide; ALT, alanine transaminase; AST, aspartate transaminase; mmHg, millimeters of mercury; mmol/L, millimoles per liter; mcL, microliter; mg/dL, milligrams per deciliter; ng/mL, nanograms per milliliter; U/L, units per liter

Laboratory test	Value	Reference range
pH	7.22	7.35-7.45
pCO2	55 mmHg	32-45 mmHg
pO2	83 mmHg	83-108 mmHg
HCO3-	20 mmol/L	
White blood cell count	15.3 x 10^3^/mcL	4.8-10.8 x 10^3 ^/mcL
Hemoglobin	14.8 g/dL	12-15 g/dL
Hematocrit	45.9%	36-47%
Platelet count	232 x 10^3^/mcL	140-450 x 10^3^/mcL
Sodium	138 mmol/L	136-145 mmol/L
Potassium	3.5 mmol/L	3.5-5.1 mmol/L
Chloride	101 mmol/L	98-107 mmol/L
CO_2_, whole blood	20 mmol/L	21-32 mmol/L
Blood urea nitrogen	24 mg/dL	7-25 mg/dL
Creatinine	2.1 mg/dL	0.7-1.3 mg/dL
Blood glucose	400 mg/dL	74- 106 mg/dL
Lactic acid	8.9mmol/L	0.5- 2.0 mmol/L
Creatinine kinase	677 U/L	39-308 U/L
Troponin	0.466 ng/mL	0-0.045 ng/mL
ALT	868 U/L	7-52 U/L
AST	629 U/L	13-39 U/L

**Figure 1:**
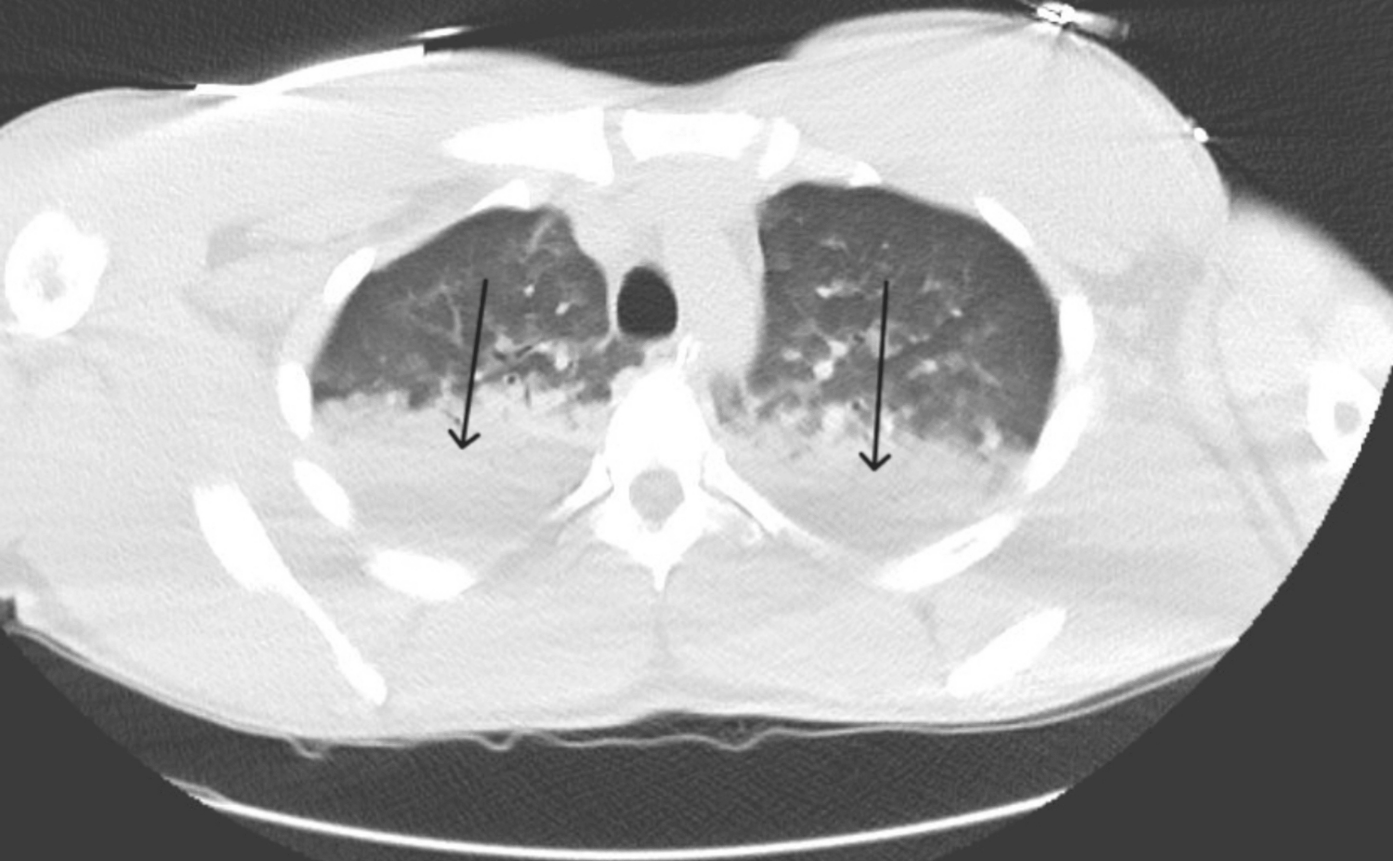
Chest CTA with arrows depicting pneumonitis. CTA, computed tomography angiography

Urine and serum drug screens were negative except for benzodiazepines. The patient’s chest computed tomography angiography (CTA) for pulmonary evaluation revealed evidence of extensive bilateral pneumonitis (Figure 1).

**Figure 2:**
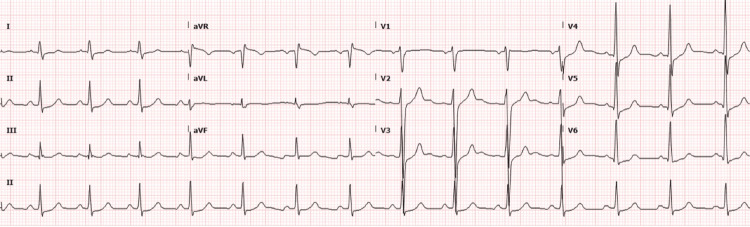
EKG performed upon arrival at 15:56. Sinus rhythm at a rate of 85 bpm. PR interval 144 ms, QRS duration 98 ms, QTc interval 410 ms. EKG, electrocardiogram

Initial electrocardiogram (EKG) revealed sinus rhythm with a rate of 85 beats per minute, normal intervals, and ST-segment elevation in lead aVR with ST-segment depression in the anterolateral leads (Figure 2).

**Figure 3:**
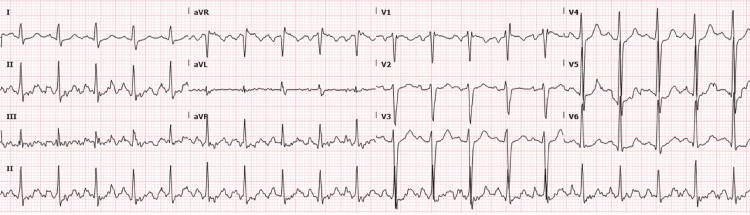
EKG performed at 16:31. Sinus tachycardia at a rate of 120 bpm. PR interval 144 ms, QRS duration 106 ms, QTc interval 411 ms. EKG, electrocardiogram

A repeat EKG revealed sinus tachycardia, a rate of 120 bpm, normal intervals, and continued ST-segment elevation in lead aVR with ST-segment depression in the anterolateral leads (Figure 3).

Rectal aspirin (324 mg) was administered. Due to concern for left main coronary artery disease based on his aVR elevation and global ST-segment depression on the EKG, emergent coronary angiography was performed by cardiology, revealing no obstructive disease but revealing an ALCA arising from the right coronary sinus. Echocardiogram performed post-catheterization demonstrated an estimated ejection fraction of 35% with moderate global hypokinesis. The patient was subsequently admitted to the intensive care unit (ICU), and therapeutic hypothermia was initiated.

**Figure 4:**
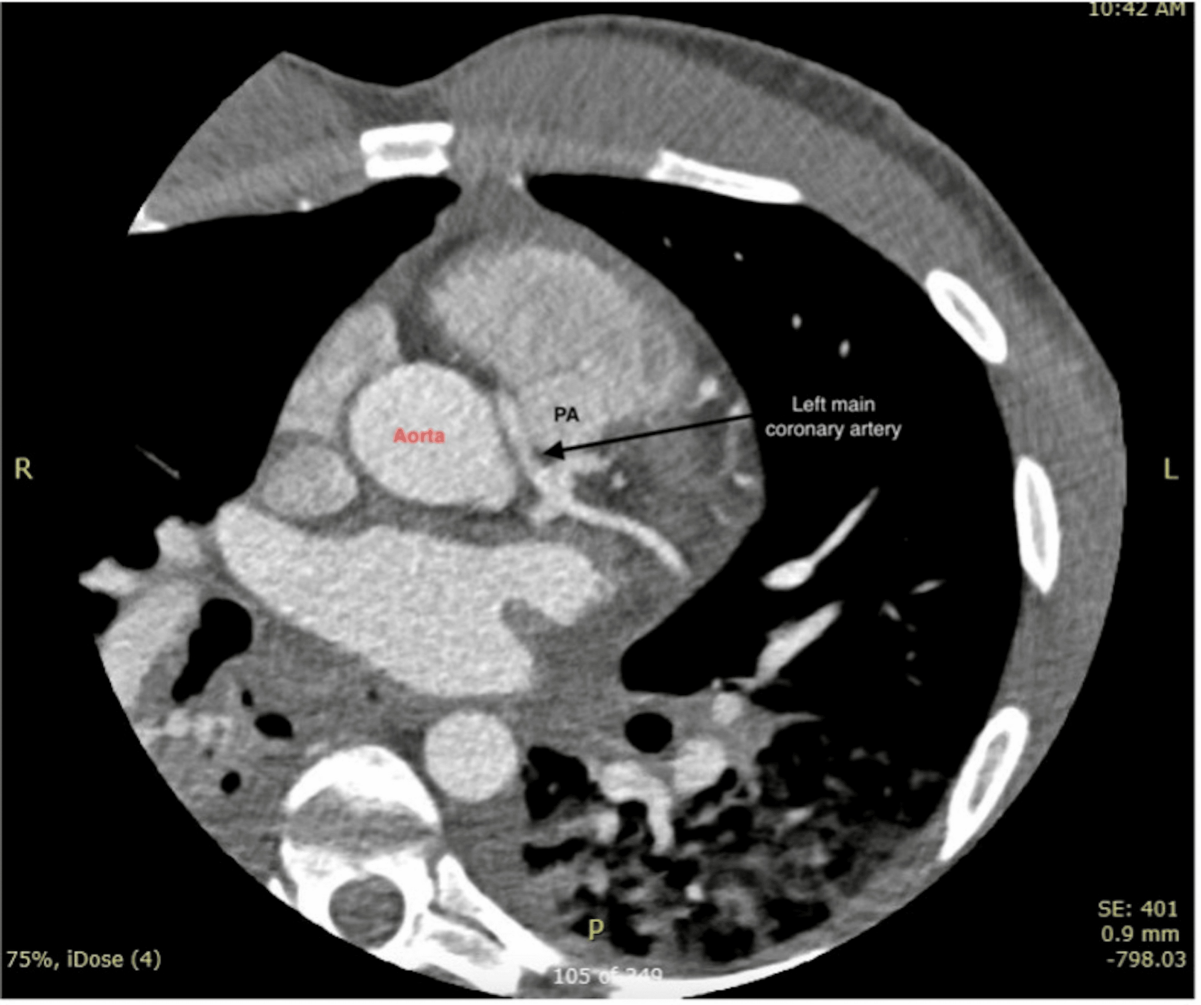
CCTA with arrows revealing the patient’s anomalous left main coronary artery coursing between the ascending aorta and main pulmonary artery. CCTA, coronary computed tomography angiography; PA, pulmonary artery

Once in the ICU, the diagnosis of anaphylaxis was ruled out, given the patient's clinical presentation and findings on the coronary angiography. The pneumonitis identified on the chest CTA was presumed secondary to the patient's intubation and cardiac arrest. On day three of the patient’s admission, a repeat echocardiogram revealed recovery of the patient’s ejection fraction to 55%-60%. The patient was extubated on day 6 and was neurologically intact. For further anatomical detail of the patient's coronary arteries, a coronary CTA was completed on the seventh day of admission and revealed an ALCA that originated from the right coronary sinus, which took an interarterial course between the ascending aorta and main pulmonary artery (Figure 4). Discussion with the patient's family revealed he had no known risk factors for coronary artery disease, nor any genetic predisposition for coronary anomalies. 

After 13 days in the hospital, the patient was discharged from the hospital with a wearable defibrillator and strict instructions to avoid all strenuous activity. He was scheduled to follow up outpatient visit with a cardiothoracic surgeon for surgical management of his coronary artery anomaly.

## Discussion

Typically, coronary arteries have the left coronary artery and right coronary artery arising from the left and right coronary sinus, respectively. Coronary artery anomalies are the anatomic aberrations of the coronary arteries from their usual course. Coronary artery anomalies are uncommon, mostly found incidentally during coronary angiography, but serve as a leading cause of sudden death amongst young competitive athletes less than 35 years of age [2,5]. Many patients with coronary artery anomalies remain asymptomatic, while others experience syncope, dyspnea, angina, or a history of aborted sudden death [6,7]. SCD can be seen in anomalies such as ectopic coronary artery origin from the pulmonary artery or the ectopic origin of the left coronary artery from the right sinus of Valsalva, as these anomalies alter blood flow to the myocardium [2]. ALCA arising from the right coronary sinus can have a significant impact on hemodynamics, with a 59% association with SCD and 81% of deaths occurring during vigorous exertion [1]. While cases of coronary artery anomaly have been reported, the causes are controversial. The interarterial course, as seen in this case, predisposes the patient to a high risk of SCD, especially among young athletes [6]. One proposed explanation is that the relative path between the aorta and pulmonary trunk puts the coronary artery, the left main coronary artery in our patient, at risk of being compressed between the two vessels during vigorous physical exertion, leading to myocardial infarction or ischemia [6,8]. This most likely occurs due to the increasing size of the aorta or pulmonary artery, and when accompanied by increased adrenergic surge, mechanical constriction of the anomalous coronary artery can occur [9,10].

Diagnosing coronary artery anomalies is challenging due to rarity and lack of prodromal symptoms in up to 93% of patients before SCD [7]. There is currently no standardized protocol to risk-stratify patients with ischemia related to coronary artery anomalies [10]. Coronary angiography has been an effective method in detecting coronary artery anomalies, but it has low utility for screening purposes due to its high cost and invasiveness [7]. Coronary CTA is a less invasive alternative with high diagnostic accuracy and is currently the gold standard method for the study of coronary artery anomalies [9, 11]. In this case, the patient is a young, healthy man with no prior history of coronary disease or ischemic symptoms, who experienced cardiac arrest during intense physical activity. Coronary angiography and Coronary CTA confirmed an ALCA originating from the right coronary sinus coursing interarterially between the ascending aorta and the pulmonary trunk. This malignant course of the left main coronary artery, with a prevalence of 0.02% in adults, is the most uncommon type of coronary artery anomaly and unfortunately predisposed the patient to a high-risk adverse cardiac event [3,4]. The patient improved and was able to follow up with the cardiothoracic surgeon for surgical correction of his anatomical coronary anomaly which is a class 1 recommendation for all patients with ALCA from the right coronary sinus due to the high risk of SCD [4]. Surgical treatment options for anomalous coronary arteries include bypass grafting, reimplantation of the artery to its correct anatomical location, or an unroofing procedure [10]. Unfortunately, despite surgical correction, this patient remains at high risk for cardiac arrest even after surgery [11].

This case report illustrates the need for consideration of coronary artery anomalies in patients presenting with signs of cardiac ischemia or SCD, particularly young athletes. In addition, this case highlights the rarity of the ALCA arising from the right coronary sinus and its malignant course, predisposing patients to SCD. Although anomalous coronary arteries are rare, it is imperative to keep this etiology in mind to expedite medical care and improve patient outcomes. Screening strategies and standardized management protocols for all types of coronary artery anomalies remain limited, emphasizing the need for further research. 

## Conclusions

ALCA from the right coronary sinus is the most uncommon type of coronary artery anomaly and is associated with an increased risk of sudden death in people ≤35 years or younger during intense physical activity. The lack of standardized screening, diagnostic, and management guidelines leaves many individuals undiagnosed and at risk, particularly young athletes. This case highlights the importance of considering coronary artery anomalies, particularly in young athletes during intense exercise, in the differential diagnosis of SCD and the need for advanced imaging, such as coronary CTA, for accurate diagnosis. Further research will be essential to develop effective screening strategies and management protocols to improve outcomes and prevent mortality. 
